# Evaluation of human primary intestinal monolayers for drug metabolizing capabilities

**DOI:** 10.1186/s13036-019-0212-1

**Published:** 2019-11-04

**Authors:** Jennifer E. Speer, Yuli Wang, John K. Fallon, Philip C. Smith, Nancy L. Allbritton

**Affiliations:** 1 Department of Chemistry, University of North Carolina, Chapel Hill, NC 27599 USA; 2 Joint Department of Biomedical Engineering, University of North Carolina at Chapel Hill, NC 27599, USA and North Carolina State University, Raleigh, NC 27607 USA; 3 Division of Pharmacoengineering and Molecular Pharmaceutics, Eshelman School of Pharmacy, University of North Carolina at Chapel Hill, NC 27599, USA and North Carolina State University, Raleigh, NC 27607 USA

**Keywords:** Drug metabolism, CYP3A4, Stiffness, Extracellular matrix, Small intestine

## Abstract

**Background:**

The intestinal epithelium is a major site of drug metabolism in the human body, possessing enterocytes that house brush border enzymes and phase I and II drug metabolizing enzymes (DMEs). The enterocytes are supported by a porous extracellular matrix (ECM) that enables proper cell adhesion and function of brush border enzymes, such as alkaline phosphatase (ALP) and alanyl aminopeptidase (AAP), phase I DMEs that convert a parent drug to a more polar metabolite by introducing or unmasking a functional group, and phase II DMEs that form a covalent conjugate between a functional group on the parent compound or sequential metabolism of phase I metabolite. In our effort to develop an in vitro intestinal epithelium model, we investigate the impact of two previously described simple and customizable scaffolding systems, a gradient cross-linked scaffold and a conventional scaffold, on the ability of intestinal epithelial cells to produce drug metabolizing proteins as well as to metabolize exogenously added compounds. While the scaffolding systems possess a range of differences, they are most distinguished by their stiffness with the gradient cross-linked scaffold possessing a stiffness similar to that found in the in vivo intestine, while the conventional scaffold possesses a stiffness several orders of magnitude greater than that found in vivo.

**Results:**

The monolayers on the gradient cross-linked scaffold expressed CYP3A4, UGTs 2B17, 1A1 and 1A10, and CES2 proteins at a level similar to that in fresh crypts/villi. The monolayers on the conventional scaffold expressed similar levels of CYP3A4 and UGTs 1A1 and 1A10 DMEs to that found in fresh crypts/villi but significantly decreased expression of UGT2B17 and CES2 proteins. The activity of CYP3A4 and UGTs 1A1 and 1A10 was inducible in cells on the gradient cross-linked scaffold when the cells were treated with known inducers, whereas the CYP3A4 and UGT activities were not inducible in cells grown on the conventional scaffold. Both monolayers demonstrate esterase activity but the activity measured in cells on the conventional scaffold could not be inhibited with a known CES2 inhibitor. Both monolayer culture systems displayed similar ALP and AAP brush border enzyme activity. When cells on the conventional scaffold were incubated with a yes-associated protein (YAP) inhibitor, CYP3A4 activity was greatly enhanced suggesting that mechano-transduction signaling can modulate drug metabolizing enzymes.

**Conclusions:**

The use of a cross-linked hydrogel scaffold for expansion and differentiation of primary human intestinal stem cells dramatically impacts the induction of CYP3A4 and maintenance of UGT and CES drug metabolizing enzymes in vitro making this a superior substrate for enterocyte culture in DME studies. This work highlights the influence of mechanical properties of the culture substrate on protein expression and the activity of drug metabolizing enzymes as a critical factor in developing accurate assay protocols for pharmacokinetic studies using primary intestinal cells.

**Graphical abstract:**

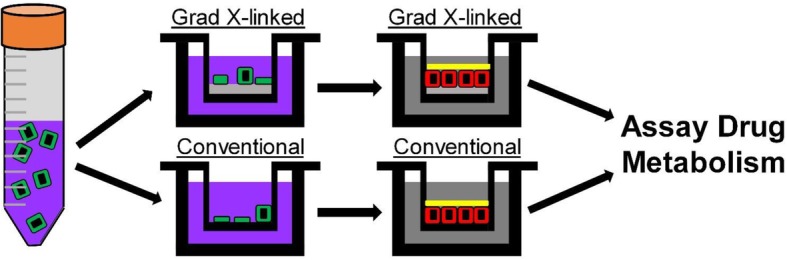

## Background

A major role of the small intestinal epithelium is to act as an initial site of nutrient and drug metabolism by enzymes within or on the surface of the enterocytes, the predominant cell type of the intestinal epithelium. These metabolic processes are supported by the small intestine’s large surface area to volume ratio enabled by a lining of villi projecting into the small intestinal lumen and further enhanced by a dense carpet of microvilli on the enterocyte surface [1]. To support their metabolic functions, enterocytes express brush border enzymes, drug transporters, and drug metabolizing enzymes (DMEs). Alkaline phosphatase (ALP) and alanyl aminopeptidase (AAP) are examples of brush border enzymes involved in the metabolism of phosphate ester-containing prodrugs or peptides [2–5]. In contrast, DMEs are typically located within the cell cytosol and internal organelles and chemically modify molecules only after they have been transported across the enterocyte membrane. Phase I DMEs convert a parent drug to a more polar metabolite by introducing or unmasking a functional group. Phase II DMEs form a conjugate between the parent compound (or metabolite) and an endogenous moiety such as glucuronic acid [6, 7]. Phase I DMEs include cytochrome P450s (CYPs) and carboxylesterases (CES) and phase II DMEs comprise enzymes such as uridine diphosphate-glucuronosyltransferases (UGTs) [8, 9]. Among these enzymes, cytochrome P450 3A (CYP3A4 in humans) and CES2 are major enzymes involved in intestinal contributions to first-pass oxidation and hydrolysis, respectively, of a variety of xenobiotics and endogenous compounds [10], while UGTs are involved in glucuronidation, a Phase II process that can also contribute significantly to intestinal first-pass metabolism. All of these enzymes play major roles in drug metabolism, and/or excretion [1].

Given the important role of the small intestine in first-pass drug metabolism and bioavailability, many model systems have been developed to simulate the interaction between the small intestine and chemical compounds, both nutrients, drugs and other xenobiotics. In vivo animal models are commonly used for drug metabolism studies; however, animal models are costly and low in throughput [11, 12]. The different animal species also possess distinct metabolic pathways, form different drug metabolites, and experience distinct drug toxicity compared to in humans [12–14]. Thus, these model systems frequently fail to recapitulate many features of the human small intestine with respect to dug metabolism, efficacy, uptake, bioavailability and toxicity. Ex vivo models utilize intact sections of intestinal tissue, often placed into perfusion systems but again are generally limited to non-human tissue [15–17]. However, both the luminal and basal environment of the small intestine is readily accessed in these models and regional intestinal differences can be studied. The loss of a blood or nutrient supply creates a short-lived tissue making this model not feasible for studying enzyme induction, drug metabolism, and drug bioavailability for more than 24 h [18]. Tissue-cultured cell lines derived from human intestinal tumors have been widely used as model systems to monitor the interaction of cells and drugs in the small intestine. Cell lines such as Caco-2 cells are easily cultured at low cost, have been extensively characterized for use in the pharmaceutical industry, and display some features representative of the normal intestinal epithelium [12, 19]. Unfortunately, Caco-2 cells have been reported to have absent or low gene expression of many phase I and II DMEs such as CYP3A4, CES2, UGT1A1, UGT1A10 and others [20, 21]. Use of tumor cell lines, even if human-derived, has in many cases led to inaccurate estimates of drug bioavailability with notable examples being antihypertensive drugs, such as verapamil and propranolol [22]. Other disadvantages of this model include the inherent chromosomal instability of tumor cells and the long differentiation period required to convert the cells into a more differentiated enterocyte-like cell for assays [23].

To address the limitations imposed by animals, ex vivo tissues and cultured tumor cells, primary human intestinal tissue derived from stem cells is increasingly used to construct model systems for study of intestinal drug metabolism. Epithelial stem cells obtained from the small intestine, cultured within Matrigel, and supplemented with appropriate growth factors form organoids resembling miniature gut tissue *aka* enteroids [24]. Enteroids demonstrate many features of the small intestinal epithelium including formation of all differentiated cell types found in vivo including enterocytes [25]. However, their spherical architecture with enclosed luminal surface and their hydrogel-embedded format makes addition to or measurement of drugs and nutrients on the luminal epithelial surface extremely challenging. For this reason, the enteroids have not become widespread for the study of drug transport and metabolism. Cell monolayers derived from primary intestinal epithelium have been developed to address the shortcomings of the enteroid culture system. These monolayers form a tight contiguous, polarized cell layer with readily accessible luminal and basal surfaces making these models suitable for drug transport and metabolism assays [26–28]. Prior studies have demonstrated that the primary monolayers also replicate many physiologic functions of the in vivo intestinal epithelium such as nutrient transport, immunologic function, chromosomal stability, barrier function, and proliferative and differentiated cell types [21, 26, 28–31]. These models have also shown CYP3A4 and CES2 gene expression and activity comparable to that found in vivo [21, 28]. However, these models are expensive and difficult to engineer, as they require three dimensional printers, flow systems and vacuums, or differentiation of primary induced pluripotent stem cells (iPSCs) several weeks in advance of the assay [21, 28, 30]. For example, a Small-Intestine-On-a-Chip required parallel culture chambers with flow and vacuum lines adding significant complexity to the culture format [30]*.* To maximize retention of in vivo drug-metabolizing enzyme activities, the enterocytes and mucosa can be isolated from human small intestines and directly cryopreserved as multicellular fragments that retain viability and function [32–34]. Although this method requires a significant amount of intestinal tissue and only supports a culture duration of 24 h, the cells do retain robust cytochrome P450 (P450) and non-P450 drug-metabolizing enzyme activities making the method valuable as an experimental model for the evaluation of enteric drug properties. Small intestinal enterocyte-like cells can be differentiated from human induced pluripotent stem cells (hiPS) and used for drug absorption and metabolism studies; however, the protocol requires 28 days and the expression patterns of hiPS derived cells are fetal-like [35*–*37]. A system that is facile to construct and utilize would significantly enhance performance of drug activity assays and be of high utility for investigating drug metabolism in vitro using primary patient samples.

In this work, we investigate two previously described simple and customizable scaffolding systems on the impact of drug metabolism by primary, human, small intestinal epithelium with a comparison to in vivo human small intestine [38]. Cells cultured as a monolayer on a gradient cross-linked scaffold or a conventional scaffold were assessed for their ability to synthesize drug metabolizing proteins as well as to metabolize exogenously added commonly used drugs. The gradient cross-linked scaffold consisted of a soft collagen hydrogel (1.2 mm thick, stiffness of 230 ± 140 Pa) that closely resembles the in vivo intestinal properties (640 ± 340 Pa) [38, 39]. The conventional scaffold was comprised of a very thin collagen film (< 900 nm) overlying a stiff surface (net stiffness of 1.50 MPa ± 0.27 MPa) [38]. In prior experiments, the monolayers in the two scaffolds demonstrated differing abilities to transport drugs, with the gradient cross-linked scaffold monolayer most closely resembling in vivo intestinal drug transport relative to that of the conventional scaffold monolayer [38]. Thus, it is possible that the scaffold monolayers may also demonstrate significantly differing capacities for drug metabolism making one more suitable for assays of human intestine-drug interactions. In this study, we investigated the ability of the two scaffolds to express relevant phase I and II DME proteins using quantitative targeted absolute proteomics by selected reaction monitoring nano-liquid chromatography-mass spectrometry/mass spectrometry (QTAP SRM nanoLC-MS/MS) techniques. The function of select phase I and II DMEs on the two scaffolding systems was also assessed. The activity of brush border enzymes involved in drug metabolism, ALP and AAP, were additionally evaluated utilizing colorimetry and fluorometric techniques. Yes-associated protein (YAP) (a key signal transducer of extracellular matrix properties) signaling was studied to evaluate whether the mechanical properties of the substrate influenced expression and function of DMEs [40].

## Results and discussion

**Fig. 1:**
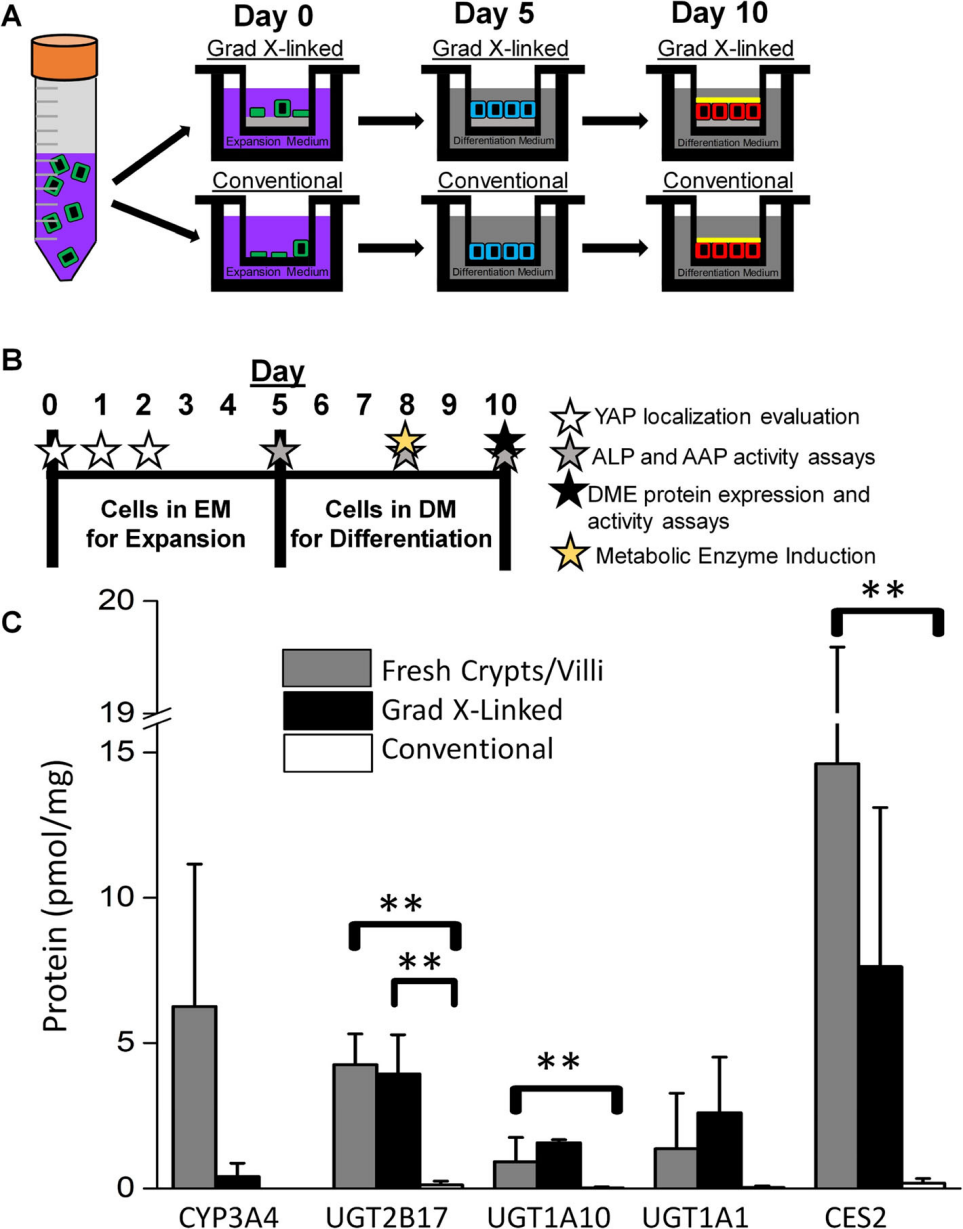
Overview of two scaffold systems used for metabolic assays. **a** Schematic of the culture conditions. Proliferative cells (green) were harvested and placed onto the two scaffolds in a growth-factor rich medium (purple, expansion medium). When the monolayer became confluent (blue cells) at day 5, the culture medium was exchanged for a medium without added intestinal growth factors (grey, differentiation medium). By day 10, the cells were differentiated and nonproliferative (red) and suitable for metabolic activity assays and possess a brush border i.e. microvilli (yellow). **b** Summary of the timing of the various assays performed in the study. **c** QTAP SRM quantification of the protein concentration (pmol of DME protein/mg of total cell protein) in the monolayer cells cultured on the gradient cross-linked and conventional scaffolds at day 10 of culture or in cells isolated from fresh crypts/villi. Shown is the average and standard deviation of the data (*n* = 3)

While the two scaffolds possess many distinctions, a major difference is that of the gradient cross-linked scaffold which possesses a stiffness similar to that of the in vivo intestine, whereas the conventional scaffold displays a stiffness several orders of magnitude greater than that of native intestine, a stiffness more characteristic of fibrotic tissue [38, 39]. Both culture systems support growth of cell monolayers covering the scaffold with cells forming an impermeable barrier to small molecules. For both monolayer systems, cells were harvested from culture, dispersed and then plated onto the different scaffolds on day 0 (Fig. 1a, b). Since the cells did not yet form a contiguous monolayer, they were cultured in medium rich in growth factors (EM, expansion medium) until day 5 when confluency was reached. The supernatant above the cells was then replaced with medium low in growth factors to assist in cell differentiation to mature enterocytes, the major cell type participating in drug metabolism. Prior work demonstrated that at day 10 of this culture protocol, the monolayer formed on both scaffolds, was impermeable to small polar molecules, comprised largely of polarized enterocytes, and displayed functional properties such as drug transport resembling that of native small intestinal epithelium [38].

### Measurement of expression of select proteins involved in drug metabolism

CYP3A4, UGT1A1, and CES2 gene expression has been evaluated in numerous primary intestinal models [21, 28, 31]. However, CYP3A4, UGTs 2B17 1A1 and 1A10, and CES2 protein expression in these primary human intestinal culture systems has not been investigated [21, 28, 30]. To assess the presence of metabolic enzyme protein within cells, QTAP SRM nanoLC/MS-MS analysis was performed on monolayers cultured for 10 days on the gradient cross-linked scaffold or conventional scaffold and compared to that of freshly isolated human crypts/villi (*n* = 3). UGTs 2B17 and 1A10 protein expression by the cell monolayers on the gradient cross-linked scaffold was significantly greater than that expressed by cells on the conventional scaffold (Fig. 1c). However, expression of these proteins and others including CYP3A4, UGT1A1 and CES2 by the cells on the gradient cross-linked scaffold was not statistically different from that of the fresh crypts/villi. The cells on the conventional scaffold also expressed similar CYP3A4 and UGTs 1A1 and 1A10 protein amounts to those found in the fresh crypts/villi but expressed statistically lower UGT2B17 and CES2 amounts to those found in the fresh crypts/villi. These data suggest that the microenvironment created by the gradient cross-linked might be more reflective of the native intestinal microenvironment needed to support drug metabolism.

### Measurement of CYP3A4 activity

**Fig. 2:**
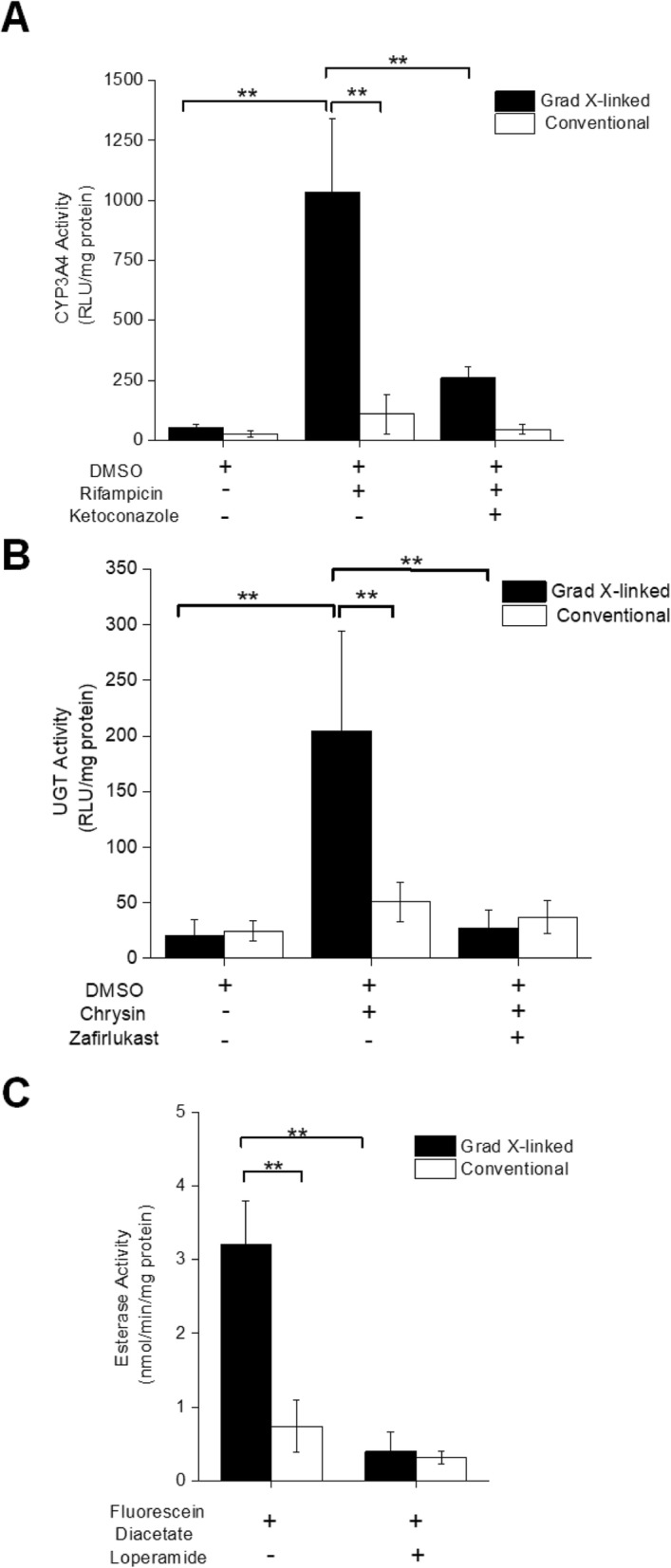
Assessment of DME activity in cells cultured on the monolayer platforms. **a** CYP3A4 activity was measured in the monolayers with and without CYP3A4 induction by prior culture in the presence of rifampicin. Rifampicin-induced cells were also assayed in the presence of the CYP3A4 inhibitor ketoconazole. Activity was reported as relative light units (RLU) per mg of protein formed over the luciferase reaction time (150 min) for panels **a** and **b**. **b** UGT activity was measured in the monolayers with and without UGT induction by prior culture in the presence of chrysin. Chrysin-induced cells were also assayed in the presence of the UGT1A10 inhibitor zafirlukast. **c** Esterase activity was measured in the monolayers with and without the CES2 inhibitor loperamide. Shown is the average and standard deviation of the data (*n* = 4)

CYP3A4 is a phase I DME that functions to metabolize drugs, typically by oxidation, and is critical for metabolism of drugs such as efavirenz and phenobarbital [41, 42]. Cells on the two scaffolds displayed CYP3A4 activity that was not statistically different (Fig. 2a) and lower than that found in other reported primary intestinal monolayers [21, 28]. However, when the monolayers on the gradient cross-linked scaffold were treated with rifampicin, an antibiotic that is a nonspecific substrate for CYP enzymes [43, 44], the CYP3A4 activity increased greatly, resulting in a fold change of 20. This fold change is significantly greater than that previously reported in other model systems, suggesting that CYP3A4 is metabolically active and binds to appropriate substrates. Caco-2 cells do not express CYP3A4 unless they are gene-engineered [19, 23, 45]. Treating the cells with rifampicin on the conventional scaffold did not significantly alter CYP3A4 activity (Fig. 2a). Ketoconazole is a known inhibitor of CYP3A4 [46] and significantly inhibited this enzyme’s activity in cells cultured on the gradient cross-linked scaffold (and pre-exposed to rifampicin). These results are similar to those reported in human primary intestinal models that require a three dimensional printer to construct the model intestinal system utilized for CYP3A4 metabolomic studies [28]. Ketoconazole did not significantly impact the cells on the conventional scaffold likely due to the absence of functional CYP3A4 protein. More than half of all drugs or their metabolites are acted upon by CYP3A4 [47], suggesting that the gradient cross-linked scaffold may be an invaluable model for testing the metabolism of a plethora of clinically relevant compounds.

### Measurement of UGT activity

The UGT family of enzymes, including UGTs 1A1, 1A10 and 2B17, are involved in the metabolism of various drug molecules in the human body and catalyze the transfer of glucuronic acid to a drug. The increase in hydrophilicity of the glucuronidated drug enables it to be more readily excreted [48]. While the mass spectrometry (MS) studies indicate detectable protein presence, the assays do not report enzyme functionality and may miss proteins present at low copy number in cells. Therefore, it was important to determine whether the enzymes in the cell monolayers were also functional. UDP glucuronosyltransferase (UGT) activity was assayed by measuring the metabolism of a UGT multienzyme substrate that is glucuronidated by UGTs 1A1 and 1A10. UGT activity in both monolayer culture systems was detectable. Chrysin, a flavonoid present in honey, is a known substrate of UGTs and upregulates their expression [48, 49]. When the cells were pre-incubated with chrysin, UGT activity increased greatly in the monolayers on the gradient cross-linked scaffold. UGT activity in the cells on the conventional scaffold remained the same with and without treatment of chrysin (Fig. 2b). Caco-2 cells have shown a 3.8 fold increase in UGT metabolite formation when treated with chrysin for 4 days [49]. The monolayers in the gradient cross-linked scaffold demonstrated a 10-fold increase in UGT activity when treated with chrysin for 2 days. Chrysin-induced UGT activity was successfully inhibited with zafirlukast, a drug commonly used to treat asthma and a known inhibitor of UGT1A10 [50, 51], in the monolayers on the gradient cross-linked scaffold (Fig. 2b). Overall, the monolayers on the conventional scaffold failed to have a statistically significant difference in UGT activity with and without the presence of a selected inducer and inhibitor, suggesting that the enzyme, if present, may not have been expressed properly or was present at low concentrations under all conditions. In contrast, the monolayers on the gradient cross-linked demonstrated significant UGT activity upon enzyme induction. The absence of activity prior to induction was most likely due to the limited sensitivity of the employed glucuronidation assay. Additionally, UGT activity was significantly diminished in the presence of a selected inhibitor. These results suggest that the gradient cross-linked scaffold is a more suitable system than the conventional scaffold for evaluating phase two UGT metabolism in primary human intestinal cells.

### Measurement of CES2 activity

CES2 is a carboxylesterase that facilitates intestinal clearance of many drugs by the hydrolysis or transesterification of a drug or drug metabolite. CES2 preferentially hydrolyzes a wide variety of endogenous esters, ester-containing drugs and environmental toxicants, such as flutamide and fluorescein diacetate [52]. To assess whether esterase activity was present within the cultured monolayers, and the relative amount, monolayers cultured on the gradient cross-linked and conventional scaffold were incubated with fluorescein diacetate and fluorescein fluorescence measured. Cells on the gradient cross-linked scaffold demonstrated significantly greater esterase activity than that on a conventional scaffold (Fig. 2c). The reported esterase activity in the monolayers on the gradient cross-linked scaffold was similar to that reported in intestinal epithelial cells derived from human iPSCs [21]. The esterase activity reported in the monolayers on the conventional scaffold was similar to that reported in Caco-2 monolayers [21]. The anti-diarrheal medication loperamide has been reported to be a non-selective inhibitor of CES2 [53, 54]. When cells cultured on the gradient cross-linked scaffold were pre-incubated with loperamide, the de-esterification of fluorescein diacetate was significantly reduced suggesting that the increased esterase activity of these cells was due to CES2. Cells cultured on the conventional scaffold were not significantly impacted by loperamide potentially due to either their already low esterase activity or lack of CES2 expression. Cells on the gradient cross-linked scaffold were assayed for CES2 activity at day 10 of culture but may express CES2 at much earlier times. In contrast the intestinal epithelial cells derived from iPSCs required 20 days of culture prior to measurement of CES2 activity. These results suggest that the cell monolayers formed on the gradient cross-linked scaffold system are suitable for assays of CES2 actions on or inhibition by various drugs.

### Measurement of brush border enzymes involved in drug metabolism

**Fig. 3:**
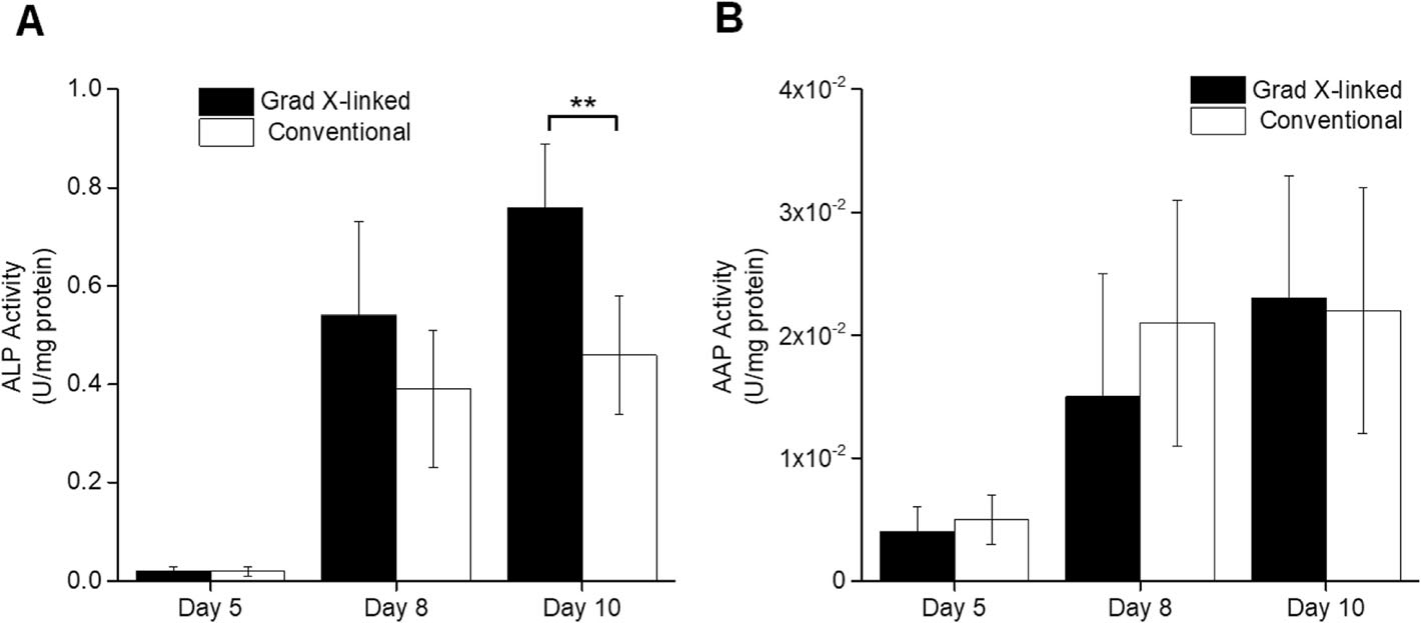
Evaluation of brush border enzyme activity. ALP (**a**) and AAP (**b**) activity of cells cultured on the gradient cross-linked and conventional scaffold on days 5, 8 and 10 of culture (*n* = 3). U = μmol/min/mL. Shown is the average and standard deviation of the data (*n* = 3). In panel (**a**), 1 U is equal to the concentration (mM) of pNPP hydrolyzed after 60 min. For AAP activity, 1 unit is equal to the μM of L-Ala-NA hydrolyzed to L-alanine and p-nitroaniline per minute

Intestinal ALP and AAP are enzymes localized to the brush border of enterocytes in the intestinal epithelium. ALP is frequently involved in the metabolism of phosphate ester pro-dugs, for example, converting the pro-drug fosamprenavir to its active antiviral form, amprenavir which is more readily absorbed than fosamprenavir [5]. AAP is a zinc exopeptidase cleaving at the amino terminal peptide end and hydrolyzing a variety of peptides but with a preference for hydrophobic and neutral residues [4]. Thus, it acts as a major barrier to the use of unmodified peptides as therapeutic compounds owing to its rapid degradation of peptides within the intestinal lumen [2, 4, 55]. The activity of ALP and AAP in cells cultured on the two scaffold systems was quantified to determine whether these monolayers might act as suitable substrates for ALP and AAP-based drug metabolism. ALP and AAP activity increased over time for the cells on both scaffold systems between days 5 and 10 suggesting that in both systems the enterocyte cell number and/or maturity increased after withdrawal of the growth-factor rich medium (Fig. 3a, b). The measured AAP activity was similar for cells on the two scaffolds whereas the cells on the gradient cross-linked scaffold displayed significantly more ALP activity at day 10 compared to the cells grown on conventional scaffolds (Fig. 3a, b). Both the ALP and AAP activity appeared comparable to that previously reported by Caco-2 monolayers on a stiff polycarbonate surface [3]. These data suggest that expression of these brush border enzymes is more robust and less dependent on scaffolding properties than that of the intracellular metabolic enzymes.

### Measurement of YAP expression and localization

**Fig. 4:**
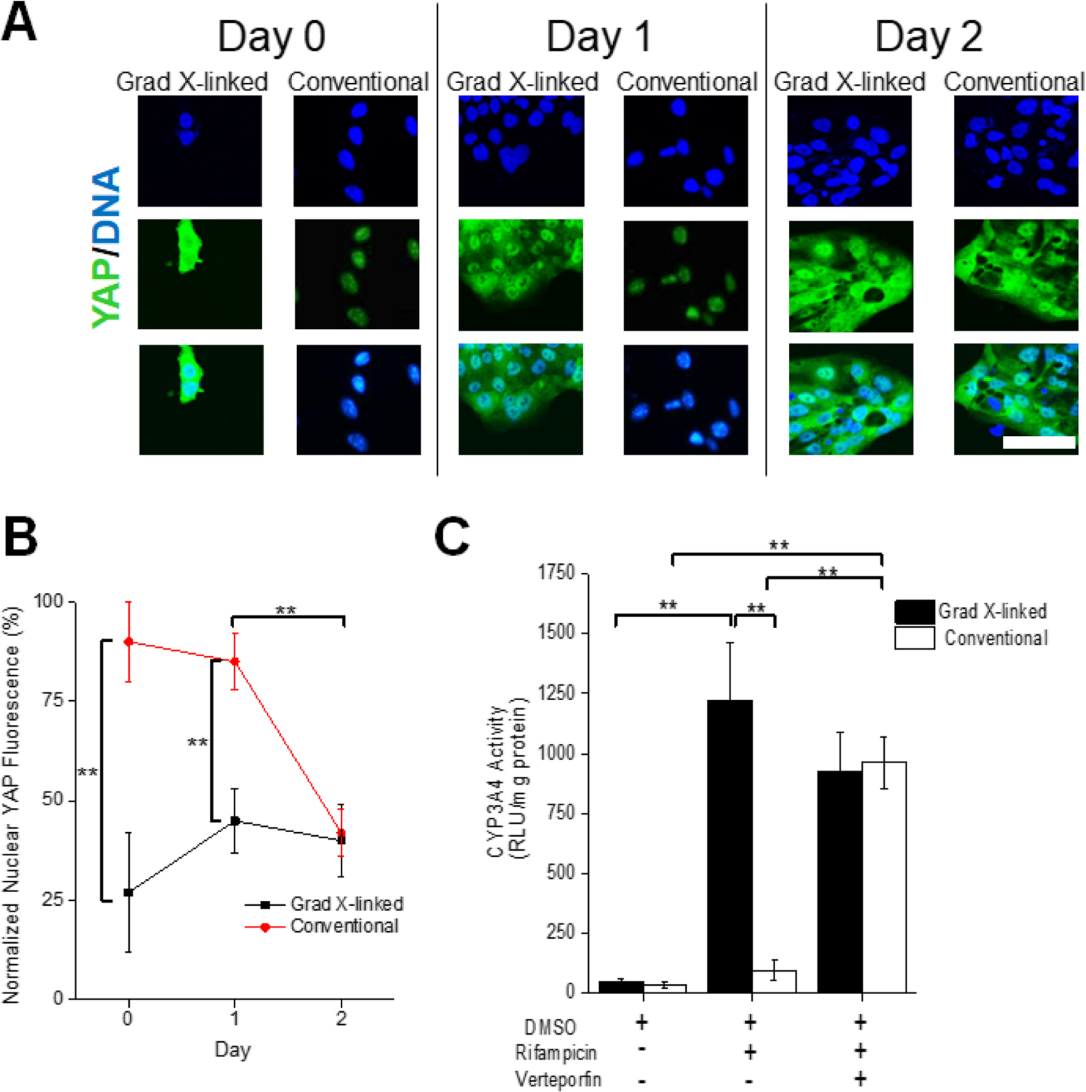
Impact of stiffness on YAP behavior. **a** YAP immunofluorescence (green) of monolayers at days 0, 1 and 2 after placement of the cells on the gradient cross-linked or conventional scaffold. DNA was counterstained with Hoechst 33342 (blue). Scale bar = 50 μm. **b** Quantification of the average fluorescence of YAP per cell nucleus for cells on the gradient cross-linked or conventional scaffold at days 0, 1 and 2 of culture. **c** CYP3A4 activity was measured for monolayers at day 10 (*n* = 4) after the cells were treated with verteporfin (YAP inhibitor) from days 0–2 and with rifampicin (CYP3A4 inducer) from days 8–10. Shown is the average and standard deviation of the data (*n* > 3)

YAP is a critical component in the mechano-transduction pathway relaying signals regarding environmental stiffness from the plasma membrane into the nucleus where YAP binds to members of the TEAD family of transcription factors in order to regulate gene transcription [40, 56, 57]. Gene transcription subsequent to YAP translocation enables the cell to respond to an altered mechanical microenvironment. To understand whether the two different scaffolds yielded altered mechano-transduction signals, YAP subcellular localization was measured at days 0, 1, and 2 after placement of cells on the scaffolds. At day 0 and day 1 after culture on the conventional scaffold, YAP was located almost exclusively within the nucleus of all cells on the conventional scaffold, while the cells on the gradient cross-linked scaffold displayed statistically significant lower nuclear YAP fluorescence signal on day 0 and day 1 than that displayed in the conventional scaffold (Fig. 4a, b). The cells on the gradient cross-linked scaffold did not display nuclei with statistically different YAP expression on the different days. These results are similar to that reported for primary intestinal enteroids cultured on a soft ECM (300 Pa) of similar stiffness to that of the gradient cross-linked scaffold [25]. The percentage of nuclei with YAP expression declined significantly on day 2 of culture in the cells on the conventional scaffold, resulting in a similar amount of nuclear YAP expression to that of the cells on the gradient cross-linked scaffold. Primary intestinal enteroids placed into a stiff microenvironment (> 1 kPa) also demonstrate a similar temporal change in nuclear YAP [25].

Since mechano-transduction signaling impacts expansion and differentiation into the absorptive cells [40, 56, 58] which express DME proteins, YAP signaling was blocked with verteporfin and the impact on CYP3A4 activity was measured. Verteporfin was added to monolayers immediately upon placement of cells on to the gradient cross-linked scaffold and conventional scaffold and maintained in the medium for 2 days. After day 2, the YAP inhibitor was removed and the cells cultured as described in the protocol of Fig. 1a. On day 8, both the cells on the gradient cross-linked scaffold and conventional scaffold were incubated with rifampicin for 2 days. On day 10, CYP3A4 activity in the monolayers on both culture systems was assessed. The monolayers on the gradient cross-linked scaffold did not show a statistically significant difference in CYP3A4 activity with and without verteporfin. In contrast, the monolayers on the conventional scaffold incubated with verteporfin demonstrated a statistically significant increase in CYP3A4 activity compared to unexposed monolayers (Fig. 4c). While not definitive, these results suggest that the mechanical properties of the microenvironment might play a role in regulating the expression or activity of proteins involved in drug metabolism via the YAP signaling pathway and YAP’s subsequent impact on cell differentiation. Further experiments assessing YAP expression throughout the differentiation protocol will be needed to confirm this hypothesis.

## Conclusions

A variety of intestinal primary cell monolayer systems have been developed on a plethora of substrates with widely varying properties, for example, polytetrafluoroethylene (PTFE), polycarbonate (PC), and polyethylene terephthalate (PET), polydimethylsiloxane (PDMS) and hydrogels, such as Matrigel and collagen [21, 26, 27, 29, 59, 60]. Here we investigate two previously described primary cell monolayer systems that possessed differing in ECM stiffness (among other attributes). The gradient cross-linked scaffold possesses a stiffness similar to that found in the in vivo intestine while the conventional scaffold possesses a stiffness orders of magnitude higher. Cells cultured on the gradient cross-linked scaffold have previously been shown to possess similar drug transporter protein expression to that found in vivo, as well as transporter function that is suitable for assay of many drug transporters. In contrast, cells on the conventional scaffold had diminished drug transporter protein expression compared to that found in fresh tissue and reduced or absent function of some transporters [38]. In this work, cells cultured on the conventional scaffold expressed significantly reduced protein expression of UGT2B17 and CES2 but similar expression of CYP3A4 and UGTs 1A1 and 1A10 relative to that found in fresh crypts/villi whereas the cells on the gradient cross-linked scaffold possessed similar CYP3A4, UGTs 2B17, 1A1 and 1A10, and CES2 protein expression to that found in the fresh crypts/villi. The monolayers on the gradient cross-linked scaffold also possessed readily measured CYP3A4, UGT, and esterase function in the presence and absence of known inducers, and function of these enzymes was successfully diminished with appropriate inhibitors. The monolayers on the conventional scaffold possessed little CYP3A4, UGT, or esterase function in the presence and absence of known inducers. The monolayers on the gradient cross-linked scaffold and conventional scaffold supported similar activity of brush border enzymes ALP and AAP. Thus, intracellular DMEs appear more highly impacted by the different culture surfaces than the brush border enzymes on the cell surface. The CYP3A4 activity of our monolayers is lower that that of recent enteric models using cryopreserved enterocytes and mucosa [32*–*34]. An important future goal will be to optimize the differentiation protocol for small intestinal stem cells by titrating growth vs differentiation signals such as Wnt, Notch and BMP-4 to better simulate the chemical sequence of events within the small intestine as the enterocytes form. Application of other environmental factors sensed by the epithelial cells of the small intestine such as mechanical stimulation, shear forces and/or a hypoxic luminal environment may also improve CYP3A4 activity. Optimization of these attributes for this model may enable a more similar level of CYP3A4 activity to that of fresh small intestinal tissue.

It is well established that intestinal stem cell growth and proliferation is influenced by mechanical characteristics, such as the stiffness of the ECM [61, 62]. *The subcellular localization of* YAP has specifically been reported to be modulated by cellular mechano-sensing and mechano-transduction sensing proteins [25]. YAP functions as a transcriptional co-activator to the TEAD family of transcription factors which regulate cell proliferation and apoptosis, controlling organ size in vivo and cell expansion and differentiation in vitro [40, 56]. YAP has exhibited enhanced nuclear translocation and activation in response to high ECM stiffness (> 1 kPA), whereas YAP has displayed cytoplasmic localization in systems with low ECM stiffness (< 500 Pa) in primary intestinal enteroids [25]. These results are consistent with those found in the monolayers on the soft gradient cross-linked scaffold and stiff conventional scaffold. The monolayers on the gradient cross-linked scaffold demonstrated temporally constant YAP fluorescence within nuclei following plating, while the monolayers on the conventional scaffold possessed high amounts YAP fluorescence within their nuclei immediately after sub-culture. By day two after sub-culture, cells on the two substrates possessed similar YAP fluorescence within their nuclei. Incubation with verteporfin, a YAP inhibitor, enhanced CYP3A4 activity in cells on the conventional scaffold to a similar level to that of cells on the gradient cross-linked scaffold. These results suggest that proteins, such as YAP, may have a role in the mechano-transduction pathways may also modulate the function of metabolic proteins found in differentiated enterocytes, such as CYP3A4. More in-depth studies will be needed to determine whether YAP and other mechano-sensing proteins modulate protein function during and after enterocyte differentiation. Nevertheless, these data demonstrate that development of in vitro models with extracellular matrices mimicking the properties of the in vivo intestine is of great importance when recapitulating physiological function of DMEs.

In summary, the data showed that the cross-linked scaffold was a superior substrate for formation of enterocytes responsive to CYP3A4 induction and the maintenance of UGT and CES activity. Despite the progress in understanding the variables impacting protein expression and function derived from this and our previous publication [38], further work will be needed to fully characterize and optimize the platform. The use of tissue derived from multiple intestinal regions and donors along with comparison to each donor’s fresh tissue would add tremendously to our understanding of biological variability and the capacity of the platform to accurately reproduce native tissue function. Mechanical cues are known to influence protein expression and enzymatic activity [63]. Furthermore, testing other metabolic enzyme substrates, such as midazolam for CYP3A4, would further define the activity profiles of clinically important proteins. In the current work, the in vitro tissue was not subjected to stress/strain influences and addition of such mechanical forces may serve to further optimize the platform. In spite of these limitations, the current work makes clear the importance of the culture substrate in influencing cell behavior and the impact on assay outcomes.

## Methods

### Materials

Rat tail type I collagen, Transwell inserts (0.9 cm^2^ cell culture area, 1.6 × 10^6^ pores/cm^2^), 12-well polystyrene tissue culture dishes, 12-well polycarbonate plates and Transwell inserts (1.12 cm^2^ cell culture area, 1.6 × 10^8^ pores/cm^2^) were purchased from Corning (Corning, NY). SB202190 was from Selleckchem (Houston, TX). Collagenase (type IV) was purchased from Worthington Biochemicals (Lakewood, NJ). Y-27632 and N-[N-(3,5-difluorophenacetyl)-L-alanyl]-S-phenylglycine t-butyl ester (DAPT) were purchased from ApexBio Technology (Houston, TX). Human collagen (type I) was obtained from Advanced Biomatrix (Carlsbad, CA). The ALP assay kit was from AbCam (Cambridge, MA). Gastrin was from Anaspec (Freemont, CA). UGT-Glo™ Assay and P450-Glo™ CYP3A4 Assay were obtained from Promega (Madison, WI). *N*-acetyl cysteine was purchased from MP Biomedicals (Santa Ana, CA). Murine EGF was procured from Peprotech (Rock Hill, NJ). Primocin was purchased from InvivoGen (San Diego, CA). Nicotinamide, L-alanine p-nitroanilide, fluorescein diacetate, loperamide, zafirlukast, cobalt chloride, potassium phosphate buffer, bovine serum albumin, and A83–01 were acquired from Sigma-Aldrich (St. Louis, MO). All other reagents and Bicinchoninic Acid (BCA) Protein Assay Kit were purchased from Thermo Fisher Scientific (Waltham, MA).

### Cell culture media composition

Expansion media (EM) for cell culture contained advanced DMEM/F12 medium, L-WRN conditioned medium, *N*-acetyl cysteine (1.25 mM), EGF (50 ng/mL), B27 (1 ×), GlutaMAX (2 mM), primocin (100 μg/mL), prostaglandin E2 (10 nM), gastrin (10 nM), nicotinamide (10 mM), HEPES (10 mM, pH 7.5), penicillin (100 unit/mL), streptomycin (100 μg/mL), SB202190 (3 μM), and Y-27632 ROCK inhibitor (10 μM). After 48 h of culture, Y-27632 ROCK inhibitor (10 μM) was removed from the EM medium used for culture. L-WRN conditioned medium was prepared by culture of L-WRN cells (ATCC #CRL-3276) as previously published [64]. L-WRN conditioned media contains Wnt-3A, R-spondin 3, and noggin at concentrations of 45, 25, and 25 ng/mL, respectively. Differentiation media (DM) contained DMEM/F12 medium, *N*-acetyl cysteine (1.25 mM), EGF (50 ng/mL), GlutaMAX (2 mM), HEPES (10 mM, pH 7.5), A83–01 (500 ng/mL), DAPT (20 ng/mL), and primocin (100 μg/mL).

### Preparation of gradient cross-linked scaffold

The gradient cross-linked scaffolds were prepared as previously described [38, 60]. Briefly, collagen (200 μL, 1 mg/mL, Type 1) was added to the upper reservoir of a Transwell insert (BD Falcon #353180, PET membrane, 0.9 cm^2^ cell culture area, 1.6 × 10^6^ pores/cm^2^), and the inserts were immediately incubated at 37 °C for 1 h. Phosphate buffered saline (PBS, 1 mL) was added to the upper and lower reservoir of each Transwell. The Transwell inserts and plates were stored at 4 °C for 30 min. The PBS in the lower compartment was immediately removed and replaced with a cocktail (1.5 mL) of 1-ethyl-3-(3-dimethylaminopropyl) carbodiimide hydrochloride (EDC, 353 mM, 500 μL) and *N*-hydroxysuccinimide (NHS, 88 mM, 500 μL) in 2-morpholinoethanesulfonic acid (MES, pH 5.5). The inserts and plates were placed at 4 °C for 40 min, followed by immediate removal of the Transwells for incubation in deionized water for 24 h to remove unreacted cross-linker. The inserts were then sterilized with 75% ethanol, washed with PBS three times, and stored in PBS at 4 °C until cell culture.

### Conventional scaffold preparation

The conventional scaffold was prepared as previously described [38]. Collagen (100 μL, 0.1 mg/mL, Type 1, < 900 nm thick) in ethanol (70%) was added to the upper reservoir of a Transwell cassette (Corning Life Sciences #3401, 1.12 cm^2^ cell culture area, 1.6 × 10^8^ pores/cm^2^). The Transwell inserts and plates were dried inside a laminar flow hood for 3 h and sterilized under UV light (280 nm) for 1 h. The upper reservoir and lower reservoir of the Transwells were immediately coated with collagen (1 mL, 0.01 mg/mL solution, Type 1) in sterile PBS. The plates were incubated in 37 °C for 24 h. The upper and lower reservoirs of the Transwell were washed with PBS three times, and the cells were plated immediately.

### Primary human small intestine monolayer cell culture

Human jejunal intestinal tissue was obtained from a single donor at UNC Hospitals under UNC IRB #14-2-2013. This tissue was used for all experiments in this manuscript. The intestinal crypts/villi were isolated from the tissue following a previously published protocol [65]. Briefly, the tissue was cut longitudinally with a scalpel to expose the luminal and basal sides of the tissue prior and then immediately incubated for 90 min at 25 °C in a 15 mL conical tube containing ethylenediaminetetraacetic acid (EDTA, 2 mM) and dithiothreitol (DTT, 0.5 mM) in buffer (5.6 mM Na2HPO4, 8.0 mM KH_2_PO_4_, 96.2 mM NaCl, 1.6 mM KCl, 43.4 mM sucrose, 54.9 mM D-sorbitol, pH 7.4). The crypts/villi were immediately detached from the tissue by vigorous shaking. All other parts of the tissue were removed from the mixture with sterile tweezers, leaving behind the crypts, EDTA, DTT, and buffer in the conical tube. The crypts/villi were isolated by centrifugation, followed by immediate removal of the supernatant and replenishment with EM. The crypts/villi in EM were plated on a collagen hydrogel (1 mg/mL, rat tail, pH 7.4, 1 mm thick) placed within a polystyrene tissue culture plate as described previously [26, 65]. The cells were passaged after 5 days following a previously published protocol [26, 65]. Briefly, the cells and collagen hydrogel were detached from the tissue culture plate’s surface with a 1000 μL pipette tip. The cells and collagen hydrogel were placed into a 15 mL conical tube containing fresh EM (1 mL) and 500 U/mL of Type IV collagenase. The collagen and cells were broken apart by repeated pipetting (1000 μL pipette tip) and then incubated at 25 °C for 10 min. The hydrogel, cells, collagenase, and EM were centrifuged at 600×g for 1 min. The isolated pellet at the bottom of the conical tube was washed with PBS (5 mL) and the mixture was centrifuged again at 600×g for 1 min, followed by immediate removal of PBS that was replaced with EDTA (0.5 mM) and Y-27632 (10 μM) in PBS. The EDTA, Y-29632, and cells were incubated at 37 °C for 10 min. The intestinal cells were re-suspended in EM (1:4) and plated on either the gradient cross-linked scaffold or conventional scaffold at a at a cell density of 1.6 × 10^5^ cells per cm^2^ as described above or stored in liquid nitrogen. In both the gradient cross-linked scaffold and conventional scaffold, the top (luminal) and bottom (basal) reservoirs contained 1 and 2 mL of EM, respectively. EM was changed to EM without Y-27632 after 48 h of culture, following EM replenishment in both reservoirs every 48 h. After the cells were cultured for 5 days, EM was removed and replaced with DM. DM was replenished every 24 h until the monolayers were utilized for assay on day 10. Cells were maintained at 37 °C in a humidified 5% CO_2_ incubator. Cells were not used for experiments beyond P15 (passage 15).

### Measurement of protein expression

Monolayers formed on both culture platforms were collected on day 10 (5 days in EM + 5 days in DM) and used for quantitative targeted absolute proteomic (QTAP) nanoLC-MS/MS analysis. To insure that the monolayers were fully differentiated and competent to form a tight network of cells, only monolayers with a transepithelial electrical resistance (TEER) > 90 Ω cm^2^ or 300 Ω cm^2^ for the gradient cross-linked scaffold and conventional, respectively, were used for measurement of protein [38]. TEER was measured using an EVOM2 Epithelial Volt/Ohm meter (World Precision Instruments, Sarasota, FL) and calculated by subtracting the raw resistance measurements of the monolayers from that of the average resistance (*n* = 3) of the collagen layer without cells and normalized by multiplying the cell culture area of the well to provide a TEER in units of Ω cm^2^ [66]. CYP3A4, UGTs 1A1, 1A10 and 2B17, and CES2 proteins were quantified using previously published methods [67, 68]. Briefly, 10 million cells were collected and homogenized in hypotonic buffer containing 10 mM tris HCl (pH 7.4), 10 mM NaCl, 1.5 mM MgCl_2_. The samples were centrifuged (10,000×g, 4 °C) and the supernatant was collected. The protein in the supernatant was isolated by ultracentrifugation (100,000×g, 4 °C) and the resulting protein pellet was submerged in fresh PBS. Quantification was enabled by addition of a stable isotope-labeled proteotypic tryptic peptide standard of known concentration (Additional file 1: Table S1). Ratios of two SRM peak area signals of the analyte summed to two corresponding signals of the stable isotope-labeled peptide standard summed were used to calculate concentrations. The equality of response between the analyte (unlabeled) and labeled peptides being assumed. The entire procedure was performed in triplicate for each platform type.

### ALP and AAP activity measurement

Intestinal cell monolayers were evaluated for ALP and AAP activity on day 5, 8, 10 of culture on the gradient cross-linked scaffold and conventional scaffold. Cell monolayers were incubated with trypsin for 10 min at 37 °C, followed by addition of EM to the collagen, cells, and trypsin. The detached cells, trypsin, and EM were placed in a 15 mL conical tube and centrifuged for 1 min at 600×g to pellet the cells. The supernatant was removed and the cell pellet washed with PBS three times, followed by resuspension in radioimmunoprecipitation assay (RIPA) lysis buffer. The mixture was homogenized by repeated pipetting with a 1000 μL tip 50 times. The supernatant was collected after the mixture was centrifuged for 15 min at 15,000×*g* at 4 °C. The supernatant was divided into 3 equal parts for measurement of ALP activity, AAP activity and total protein. Total protein was measured using a Bicinchoninic Acid (BCA) Protein Assay according to the manufacturer’s instructions (Thermo Fisher Scientific, Waltham, MA). The ALP activity was quantified according to the manufacturer’s instructions (Abcam, Cambridge, MA) using 5 mM para-Nitrophenylphosphate (*p*NPP) as substrate. ALP activity was expressed in units (U), where 1 U is equal to the concentration (mM) of *p*NPP hydrolyzed after 60 min. *p*NPP was quantified by measurement of its absorbance at 405 nm followed by comparison to a standard curve (Spectramax M5 plate reader, Molecular Devices, San Jose, CA). The AAP assay was performed following a previously published protocol [3]. Briefly, L-alanine p-nitroanilide (L-Ala-NA) was used as a substrate. Cobalt chloride (1 mM) in 10 mM tris HCL buffer (pH 8.0) was mixed with the cell lysate and incubated at 37 °C for 10 min to activate AAP. L- Ala-NA (1.66 mM) in 60 mM potassium phosphate buffer solution was immediately added to the cell lysate. After 30 min, the absorbance of the solution was measured at 405 nm and compared to that of a standard to quantify the breakdown of L-Ala-NA. AAP activity is expressed in units (U). For AAP activity, 1 unit is equal to the μM of L-Ala-NA hydrolyzed to L-alanine and p-nitroaniline per minute.

### DME functional assays

All DME activity assays were performed on monolayers with a TEER > 90 Ω cm^2^ or 300 Ω cm^2^ for the gradient cross-linked scaffold and conventional scaffold, respectively. TEER was measured before and after the assay to further verify that monolayer integrity remained constant throughout the assay.

CYP3A4 and UGT activity were assessed in the presence and absence of a known inducer. Rifampicin was used as an inducer for CYP3A4 activity while chrysin was utilized as an inducer for UGT activity. For induction of enzyme activity, monolayers were cultured with 20 μM rifampicin or 50 μm chrysin or DMSO vehicle (final concentration 0.1%) for 48 h (days 8–10). On day 10 the activity of the enzymes was measured after preincubation for 30 min with DMSO control vehicle or inhibitor (1 μM ketoconazole and 1 μM zafirlukast for CYP3A4 and UGT assays, respectively). Cells were lysed with RIPA buffer and protein quantified using Pierce BCA Protein Assay kit. Then, CYP3A4 and UGT activity was measured following the manufacturer’s instructions (Promega, Madison, WI). Briefly, CYP3A4 activity was assessed by adding luciferin-IPA to monolayers for 60 min (37 °C, 5% CO_2_). The P450 Glo reagent was added to each well and equilibrated for 20 min. UGT activity was measured by adding UGT reaction buffer, 4 mM UDPGA, and 20 μM UGT multienzyme substrate. Reactions were terminated after incubation (37 °C, 5% CO_2_) for 150 min by addition of firefly luciferase detection mixture supplemented with 20 mM D-cysteine (LDR). The luminescence was quantified using a Spectramax M5 plate reader (Molecular Devices, San Jose, CA) and reported as relative light units (RLU) per mg of protein formed over the luciferase reaction time (150 min) as recommended by the reagent manufacturer.

To evaluate if mechano-transduction signaling regarding environmental stiffness impacts expansion and differentiation into the absorptive cells which express DME proteins, YAP signaling was blocked by treating the monolayers with 500 nmol verteporfin immediately upon placement of cells on to the gradient cross-linked scaffold and conventional scaffold and maintained in the medium for 2 days. After day 2, the verteporfin was removed. On day 8, both the cells on the gradient cross-linked scaffold and conventional scaffold were incubated with rifampicin for 2 days. On day 10, CYP3A4 activity in the monolayers on both culture systems was evaluated as described above.

To measure esterase activity, the cells were lysed with buffer (50 mM Tris–HCl (pH 7.4), 150 mM NaCl, 0.5% Triton X-100, and 1 mM EDTA) and placed on ice for 20 min. The mixture was centrifuged at 15,000×g at 4 °C for 15 min, and the supernatant was collected. The protein in the supernatant was quantified using Pierce BCA Protein Assay kit. Supernatant (20 μg/mL protein) was incubated with and without 1 mM loperamide (CES2 inhibitor) at 37 °C for 5 min and then treated with 10 μM fluorescein diacetate (CES2 substrate). After 15 min of incubation, the reaction was terminated by the addition of an equal volume of ice-cold acetonitrile. The fluorescence intensity was immediately measured with a Spectramax M5 plate reader (Molecular Devices, San Jose, CA) using the wavelengths 485 nm for excitation and 535 nm for emission. Esterase activity is reported as nmol fluorescein formed per min of reaction time per mg of cell protein (nmol/min*mg).

### YAP staining and image analysis

The monolayers (*n* = 3) were stained for expression of Yes-associated Protein (YAP) on day 1, day 2, and day 3 of culture on placement of the gradient cross-linked or conventional scaffolds to evaluate YAP subcellular localization. The cell monolayers were fixed by incubation with paraformaldehyde (4%) for 20 min, washed three times with PBS, and permeabilized with 0.5% triton X-100 for 20 min at 25 °C. The monolayers were then washed twice with PBS and immediately incubated with bovine serum albumin (BSA, 3%) for 50 min at 25 °C. After blocking with BSA, the cells were incubated in primary antibody (1:50, sc-101,199, Santa Cruz Technology, Santa Cruz, California) at 4 °C for 24 h. The monolayers were washed with PBS and incubated with donkey anti-mouse secondary antibody conjugated to AF 647 fluorophore (1:300, Jackson Immunoresearch #715–605-020, West Grove, PA) for 45 min at 25 °C. The monolayers were washed three times with PBS and the DNA was stained with Hoechst 33342 (2 μg/mL). The monolayers were incubated for 15 min at 25 °C with the DNA stain and washed with PBS three times.

Images were acquired using a confocal laser-scanning microscope (Fluoview FV3000; Olympus, Waltham, MA). YAP staining was visualized with a CY5 filter (excitation filter 604–644 nm, emission 672–712 nm). DNA stained with Hoechst 33342 was viewed using a DAPI filter (excitation filter 352–402 nm, emission 417–477 nm). All images shown were acquired with a 20× objective (N.A. = 0.45). Images were empirically thresholded using Image J (https://imagej.nih.gov/ij/). Hoechst 33342 fluorescence was segmented as a marker of the location of the cell nuclei. The fluorescence of YAP within the nuclei of each sample was then quantified and reported as the average fluorescence intensity per nucleus (DNA stain) resulting in a number that expresses the percentage of the cells with nuclear YAP expression. Four different locations in the same monolayer were assessed and this experiment was repeated (*n* = 3). The average fluorescence intensity per nucleus with a single standard deviation is shown (average ± SD).

### Statistical analysis

Statistical analysis was performed using a one-way analysis of variance (ANOVA) followed by a multiple comparison test with Tukey’s honestly significant difference procedure conducted at the 5% significance level. In all figures, ‘**’ denotes *p* < 0.05. The average with a single standard deviation (SD) is shown (average ± SD). All experiments were performed in triplicate unless specified otherwise.

## Supplementary information

**Additional file 1: Table S1.** Peptide markers of all DME proteins evaluated in the monolayers and crypts/villi via QTAP SRM can be found in the supporting information.

## Data Availability

The datasets during and/or analyzed during the current study available from the corresponding author on reasonable request.
