# Supplementary material for: Evaluation of human primary intestinal monolayers for drug metabolizing capabilities
Source: J Biol Eng. 2019 Nov 4;13:82. doi: 10.1186/s13036-019-0212-1 (PMC6829970; doi:10.1186/s13036-019-0212-1)
Supplement: Supplementary file 1 — Additional file 1: Table S1. Peptide markers of all DME proteins evaluated in the monolayers and crypts/villi via QTAP SRM can be found in the supporting information. [file 13036_2019_212_MOESM1_ESM.docx]

**Additional file**

**Evaluation of Human Primary Intestinal Monolayers for Measurement of Drug Metabolism**

Jennifer E. Speer^1^, Yuli Wang^1^, John K. Fallon^3^, Philip C. Smith^3^, Nancy L. Allbritton^1, 2,^ *****

^1^Department of Chemistry, University of North Carolina at Chapel Hill, NC 27599, USA

^2^Joint Department of Biomedical Engineering, University of North Carolina at Chapel Hill, NC 27599, USA and North Carolina State University, Raleigh, NC 27607, USA.

^3^ Division of Pharmacoengineering and Molecular Pharmaceutics, Eshelman School of Pharmacy, University of North Carolina at Chapel Hill, NC 27599, USA and North Carolina State University, Raleigh, NC 27607, USA.

*Correspondence to: Nancy L. Allbritton, Email: nlallbri@unc.edu.

Joint Department of Biomedical Engineering, University of North Carolina, Chapel Hill, North Carolina 27599, USA and North Carolina State University, Raleigh, North Carolina 27695, USA.

Tel.: +1-919-966-2291; fax: +1-919-962-2388

**Table of Contents**

Table 1. Peptide markers of all DME proteins evaluated in the monolayers and crypts/villi via QTAP SRM.

**Table S1**. Peptide markers of all DME proteins evaluated in the monolayers and crypts/villi via QTAP SRM. Amino acids shown in bold are ^13^C and ^15^N labeled peptides. The mass differences between labeled (shown) and unlabeled (not shown) R, F, K and L are 10, 10, 8 and 7 respectively. Mass shift for transition ions, between labeled and unlabeled, also depends on the charge state. The product ion for most labeled peptide multiple reaction monitoring (MRMs) contains the heavy label. Where necessary, peptides used when reporting concentrations are marked with **●**.

| **Protein**  **(& Gene)** | **Peptide Sequence** | **MRM1^a,b^**  **(product ion)**  **(mass spec specific)** | **MRM2**  **(product ion)**  **(mass spec specific)** |
| --- | --- | --- | --- |
| UGT1A1 | G_54_HEIVVLAPDASLYI**R**_69_ | 588.33/472.76 (y8) | 588.33/819.47 (b8) |
| UGT1A1 | D_70_GA**F**YTLK_77_**●** | 462.75/681.39 (y5) | 462.75/524.31 (y4) |
| UGT1A1 | T_78_YPVPFQ**R**_85_ | 509.27/753.43 (y6) | 509.27/557.31 (y4) |
| UGT1A3 | Y_164_LSIPTVFFL**R**_174_ | 683.39/1089.63 (y9) | 683.39/889.52 (y7) |
| UGT1A4 | F_74_FTLTAYAVPWTQ**K**_87_ | 840.95/1000.53 (y8) | 560.97/667.37 (y5) |
| UGT1A4 | V_92_TLGYTQG**F**FETEHLLK_108_ | 665.02/869.45 (y7) | 665.02/740.42 (y6) |
| UGT1A4 | Y_164_LSIPAVFFW**R**_174_ | 704.89/1132.61 (y9) | 704.89/932.51 (y7) |
| UGT1A5 | Y_164_LSIPAVFFL**R**_174_ | 668.39/1059.62 (y9) | 668.39/859.51 (y7) |
| UGT1A6 | D_44_IVEVLSD**R**_52_ | 528.28/728.38 (y6) | 528.28/599.34 (y5) |
| UGT1A6 | S_103_FLTAPQTEY**R**_113_ | 661.83/803.39 (y6) | 661.83/874.43 (y7) |
| UGT1A7 | T_76_YSTSYT**L**EDQDR_88_ | 529.24/662.27 (y5) | 793.36/883.42 (y7) |
| UGT1A7 | W_98_TAPL**R**_103_ | 377.22/466.32 (y4) | 377.22/567.35 (y5) |
| UGT1A8 | G_52_HEVVVVMPEVSWQLG**K**_68_ | 634.68/526.29 (y9) | 634.68/720.40 (b7) |
| UGT1A9 | A_92_FAHAQW**K**_99_ | 322.84/540.31 (y4) | 322.84/341.22 (y2) |
| UGT1A9 | G_171_ILCHYLEEGAQCPAPLSYVP**R**_192_ | 847.41/1009.56 (y9) | 847.41/841.48 (y7) |
| UGT1A10 | T_76_YSTSYT**L**EDQNR_88_**●** | 528.91/661.29 (y5) | 792.87/882.44 (y7) |
| UGT1A10 | Y_160_FSLPSVVFT**R**_170_ | 663.36/815.47 (y7) | 663.36/1015.59 (y9) |
| UGT2A3 | V_41_ILEELIV**R**_49_ | 547.35/881.53 (y7) | 547.35/768.45 (y6) |
| UGT2A3 | A_319_NIIASALAQIPQ**K**_332_ | 723.44/380.25 (y3) | 482.63/380.25 (y3) |
| UGT2B4 | F_174_SPGYAIE**K**_182_ | 510.27/785.42 (y7) | 510.27/688.41 (y6) |
| UGT2B4 | A_253_DIWLI**R**_259_ | 448.77/597.35 (y4) | 448.77/486.25 (b4) |
| UGT2B7 | I_74_EIYPTSLT**K**_83_ | 586.84/654.41 (y6) | 586.84/817.45 (y7) |
| UGT2B7 | A_253_DVWLI**R**_259_ | 441.76/597.39 (y4) | 441.76/696.43 (y5) |
| UGT2B10 | G_49_HEVTVLASSASILFDPNDSSTL**K**_72_ | 832.77/869.48 (y8) | 832.77/1131.54 (y10) |
| UGT2B10 | N_259_SWNF**K**_264_ | 402.21/416.25 (y3) | 402.21/602.33 (y4) |
| UGT2B11 | G_50_HEVTVLASSASILFDPNDASTL**K**_73_ | 827.43/1115.56 (y10) | 827.43/853.46 (y8) |
| UGT2B11 | F_74_EVYPTSLT**K**_83_ | 596.83/916.53 (y8) | 596.83/654.40 (y6) |
| *Supplemental Table 1, continued****.*** | | | |
| UGT2B15 | F_175_SVGYTFE**K**_183_ | 543.28/752.38 (y6) | 543.28/851.44 (y7) |
| UGT2B15 | S_432_VINDPVY**K**_440_ | 521.79/856.47 (y7) | 521.79/743.38 (y6) |
| UGT2B17 | F_175_SVGYTVE**K**_183_**●** | 519.28/803.43 (y7) | 519.28/704.40 (y6) |
| UGT2B17 | S_432_VINDPIY**K**_440_ | 528.80/870.48 (y7) | 528.80/757.40 (y6) |
| UGT2B28 | W_356_IPQNDLLGLP**K**_367_ | 701.41/551.83 (y10) | 701.41/1102.65 (y10) |
| UGT2B28 | L_445_SIIQHDQPVKPLH**R**_459_^c^ | 448.51/532.32 (y4) | 448.51/492.80 (y8) |
| CYP1A2 | Y_244_LPNPALQ**R**_252_ | 541.31/403.23 (y7) | 541.31/594.36 (y5) |
| CYP2E1 | F_360_ITLVPSNLPHEAT**R**_374_ | 568.98/566.29 (y10) | 568.98/720.37 (y6) |
| CYP2C8 | N_466_LNTTAVT**K**_474_ | 485.28/742.43 (y7) | 485.28/628.39 (y6) |
| CYP2C19 | G_98_HFPLAE**R**_105_ | 312.84/385.21 (y3) | 312.84/439.21 (b4) |
| CYP2B6 | E_254_TLDPSAP**K**_262_ | 483.26/507.31 (y5) | 483.26/622.34 (y6) |
| CYP2B6 | G_379_YIIP**K**_384_ | 349.73/478.36 (y4) | 349.73/365.27 (y3) |
| CYP2B6 | G_110_YGVIFANGN**R**_120_ | 589.30/801.42 (y7) | 589.30/688.34 (y6) |
| CYP2J2 | L_214_LDEVTYLEAS**K**_225_ | 694.87/819.43 (y7) | 694.87/918.50 (y8) |
| CYP2J2 | D_276_FIDAYL**K**_283_ | 496.76/730.42 (y6) | 496.76/617.34 (y5) |
| CYP2D6 | S_116_QGVFLA**R**_123_ | 444.25/672.41 (y6) | 444.25/516.32 (y4) |
| CYP2D6 | D_381_IEVQGF**R**_388_ | 487.25/745.39 (y6) | 487.25/517.28 (y4) |
| CYP3A5 | D_244_TINFLS**K**_251_ | 473.30/217.10 (b2) | 473.26/729.44 (y6) |
| CYP3A4 | L_477_SLGGLLQPEKPVVL**K**_492_^c^**●** | 567.03/693.43 (y13) | 567.03/664.92 (y12) |
| CYP3A4 | L_331_QEEIDAVLPN**K**_342_ | 688.88/366.22 (y3) | 688.88/1135.61 (y10) |
| CYP2C9 | G_98_IFPLAE**R**_105_ | 456.76/595.34 (y5) | 456.76/298.18 (y5) |
| CYP2C9 | S_460_LVDP**K**_465_ | 333.70/252.18 (y2) | 333.70/466.28 (y4) |
| CYP2A6 | G_162_TGGANIDPTFFLS**R**_176_ | 781.90/877.48 (y7) | 781.90/992.51 (y8) |
| CYP2A6 | D_401_PSFFSNPQDFNPQHFLNE**K**_420_ | 806.04/811.40 (y13) | 806.04/911.93 (y15) |
| CYP4F2 | S_109_VINASAAIAP**K**_120_ | 575.35/850.50 (y9) | 575.35/665.42 (y7) |
| CYP4F2 | F_445_DPENI**K**_451_ | 435.73/608.36 (y5) | 435.73/304.68 (y5) |
| CES1 | L_172_GIWGFFSTGDEHS**R**_186_ | 573.61/718.32 (y12) | 573.61/625.28 (y11) |
| CES1 | G_187_NWGHLDQVAAL**R**_199_ | 482.92/638.34 (y11) | 482.92/545.31 (y10) |
| CES1 | F_303_LSLDLQGDP**R**_313_ | 635.84/1010.51 (y9) | 635.84/810.40 (y7) |
| CES1 | E_394_LIPEATE**K**_402_ | 519.29/682.36 (y6) | 519.29/341.68 (y6) |
| CES2 | T_35_THTGQVLGSLVHV**K**_49_**●** | 396.98/430.78 (y8) | 528.97/747.47 (y7) |
| CES2 | L_179_GVLGFFSTGD**K**_190_ | 624.84/866.42 (y8) | 624.84/979.51 (y9) |
| CES2 | E_366_ASQAALQ**K**_374_ | 477.27/538.35 (y5) | 477.27/753.45 (y7) |

^a^ Two MRM methods were used to acquire data. The highest number of MRMs in a method was 208

^b^ MRMs are roughly in order of highest intensity

^c^ KP is a missed cleavage site. P prevents trypsin cleavage at the adjacent preceding K
